# Shiga Toxin-Producing *Escherichia coli* O157 Is More Likely to Lead to Hospitalization and Death than Non-O157 Serogroups – Except O104

**DOI:** 10.1371/journal.pone.0078180

**Published:** 2013-11-14

**Authors:** Karina Preußel, Michael Höhle, Klaus Stark, Dirk Werber

**Affiliations:** 1 Robert Koch Institute, Department for Infectious Disease Epidemiology, Berlin, Germany; 2 Berlin School of Public Health, Charité - Universitätsmedizin Berlin, Berlin, Germany; Beijing Institute of Microbiology and Epidemiology, China

## Abstract

The clinical spectrum following infection with Shiga toxin-producing *Escherichia coli* (STEC) is wide ranging and includes hemorrhagic colitis and life-threatening hemolytic uremic syndrome (HUS). Severity of STEC illness depends on patients' age and strongly on the infecting strains' virulence. Serogroup O157 is often assumed to be more virulent than others. Age-adjusted population-based data supporting this view are lacking thus far.

We conducted a large retrospective cohort study among patients of community-acquired gastroenteritis or HUS diagnosed with STEC infection, reported in Germany January 2004 through December 2011. Age-adjusted risks for reported hospitalization and death, as proxies for disease severity, were estimated for STEC serogroups separately, and compared with STEC O157 (reference group) using Poisson regression models with robust error estimation.

A total of 8,400 case-patients were included in the analysis; for 2,454 (29%) and 30 (0.4%) hospitalization and death was reported, respectively. Highest risks for hospitalization, adjusted for age and region of residence, were estimated for STEC O104 (68%; risk ratio [RR], 1.33; 95% confidence interval [CI], 1.19–1.45), followed by STEC O157 (46%). Hospitalization risks for the most prevalent non-O157 serogroups (O26, O103, O91, O145, O128, O111) were consistently and markedly lower than for O157, with the highest RR for O145 (0.54; 95% CI, 0.41–0.70) and the lowest for O103 (0.27; 95% CI, 0.20–0.35). Mortality risk of O104 was similar to O157 (1.2% each), but the group of all other non-O157 STEC had only 1/10 the risk (RR, 0.09; 95% CI, 0.02–0.32) compared to O157.

The study provides population-based and age-adjusted evidence for the exceptional high virulence of STEC O157 in relation to non-O157 STEC other than O104. Timely diagnosis and surveillance of STEC infections should prioritize HUS-associated *E. coli*, of which STEC O157 is the most important serogroup.

## Introduction

Shiga toxin-producing *Escherichia coli* (STEC) are a heterogeneous group of organisms [1]–[3] and the clinical spectrum caused by gastrointestinal infection with STEC varies widely [4], [5]. Besides asymptomatic infection it includes acute non-bloody diarrheal illnesses, but also hemorrhagic colitis and the hemolytic uremic syndrome (HUS), a life-threatening thrombotic microangiopathy leading to acute renal dysfunction approximately one week after onset of diarrhea. STEC of serotype O157:H7 is the leading cause of pediatric HUS [6], [7]. Because of the pathogen's inability to ferment sorbitol within 24 hours, culture identification of this serotype is easy, reliable and inexpensive [8].

Numerous other STEC serotypes exist [9] and many have been isolated from humans [10]–[12]. In many regions, these “non-O157” STEC are at least as commonly identified as STEC of serogroup O157 (comprising H7 and, less frequently, H- serotypes) in patients with acute community-acquired diarrhea [13]–[18]. However, diagnosis of sorbitol-fermenting non-O157 STEC is complex and requires nonculture screening strategies because selective and differential media are not available for their culture [19]. The historically rooted distinction between (sorbitol-nonfermenting) STEC O157 and (sorbitol-fermenting) non-O157 STEC is still widely upheld [19]. This is probably due to these differences in diagnosis and the notion - supported by several studies - that STEC O157, on average, is more virulent than many if not all other STEC serogroups [20], [21], and is particularly outbreak-prone [22]. This notion, however, is not unequivocal [23]–[25]. The discrepant diagnostic approaches for O157 and non-O157 STEC and the frequency with which they are performed [26], [27] impede studies on serogroup-specific virulence or burden of illness in many countries that primarily use selective and differential media for identifying STEC O157. Inferences or generalizations of recent epidemiological studies of STEC infections are often hindered by convenience sampling [11], [28] or by drawing on a limited number of laboratories or a small geographic area [14]–[17], [20], [21], [29]–[32]. Furthermore, severity of STEC illness likely depends on patients' age, as indicated by the observation that the median age of STEC O157 patients with HUS is younger than those without HUS [33]. Yet, none of the sparse population-based comparisons between STEC serogroups has accounted analytically for the possible confounding effect of patients' age.

In Germany, STEC diagnosis is based on detection of Shiga toxins by immunoassays or their encoding genes by polymerase chain reaction, allowing for serogroup-independent identification of STEC. Subsequent culture isolation and serotyping is recommended but not mandatory and rarely performed in clinical laboratories. It is frequently performed by the German National Reference Center for bacterial enteric pathogens. The objective of our study was to quantify the age-adjusted risk of hospitalization and death separately for STEC serogroups, including O104 that caused a large outbreak in 2011 [34], and compare non-O157 serogroups with STEC O157 in Germany.

## Methods

### Data source

Data were extracted from Germany's national reporting database on infectious diseases, hosted by the Robert Koch Institute (RKI), Germany's federal level public health institute. In Germany, both serogroup-independent detection of STEC in stool and clinically diagnosed “enteropathic” (i.e., diarrhea-associated) HUS are reportable entities. For every notified person in Germany fulfilling the surveillance case definition (see below), a case report is filed by the local health department and transmitted (without name and address of the patient) electronically, via state health departments, to the RKI. The surveillance case definition for STEC gastroenteritis (without HUS) requires detection of Shiga toxin or their encoding genes from stool culture or detection of Shiga toxin genes from stool enrichment culture in a person with symptoms compatible with STEC-gastroenteritis, i.e., at least one of the following: diarrhea (three or more loose stools in a 24 hour period), abdominal cramps or vomiting. The case definition also includes persons with compatible clinical symptoms without laboratory-confirmation if they are epidemiologically linked to a laboratory-confirmed case (these cases represent <2%). The surveillance case-definition for HUS requires that two of the following three criteria are marked on the case report: hemolytic anemia, thrombocytopenia (platelet count ≤150,000/mm^3^), or acute renal dysfunction, defined as either oliguria (<500 ml/24 h), or proteinuria, or hematuria.

An electronic case-report includes, among other entities, information on age, sex and county of residence, hospitalization, and death of the patient. Health departments are asked to mark the field for death if the infectious disease had contributed to the death. An email is sent to the local health department with the purpose of verifying such death notices and the assessment of a (co-)causal contribution of the infectious disease. In addition, the case-report includes the following dates: disease onset, diagnosis, reporting and, where applicable, hospitalization and death.

### Study Design

We conducted a retrospective cohort study among patients with community-acquired gastroenteritis or HUS in whose stool STEC was detected, reported in Germany from January 2004 through December 2011 and for whom a report was transmitted to the RKI according to the surveillance definition. For HUS cases, evidence for an STEC infection could also be established by detecting anti-lipopolysacharide IgM antibodies against *E. coli* serogroups in blood.

We used two outcomes as a proxy for disease severity: reported hospitalization and death. Covariates besides STEC serogroup were cases' age, sex, region of residence, and season of infection. For descriptive analysis and for serogroup-specific analysis of hospitalization risks, the serogroup variable had the following categories: O157, O145, O128, O111, O104, O103, O91, O26 (the 8 most frequently reported O antigens), the group of all other detected serogroups (“other”), and the group of all cases without serogroup information (“unknown”). For a comparison of O157 with all “conventional” non-O157 STEC (i.e., all except O104), we additionally conducted an analysis with a recoded serogroup variable that combined all cases with a known serogroup, except O157 and O104, into one category. This coding was also used for analysis of serogroup-specific mortality risks. Age groups (<3 years, 3–9 years, 10–18 years, 19–40 years, 41–65 years, and >65 years) were generated based on nonlinear descriptive analysis using locally weighted regression (LOWESS, [35]), biological plausibility, and strata size. Information of cases' county of residence was aggregated to four German geographic regions of residence (North, East, South, West). Seasonality of disease was categorized into “spring” (calendar week [CW] 9–21), “summer” (CW 22–34), “fall” (CW 35–47), and “winter” (CW 48–8) generated from cases' reporting dates.

We excluded cases from all analyses if they had missing values in covariates (age, sex, residence) or the binary outcome variable. As it is impossible to assess risk of hospitalization in already hospitalized patients, we also excluded those with potentially hospital-acquired infection, i.e. if their date of admission to the hospital preceded the date of symptom onset by at least two days (the minimum incubation period for STEC O157). For hospitalized cases with missing date of symptom onset or hospitalization (n = 931), we conducted a single logistic imputation procedure, based on the relation between hospital-acquired STEC- infection and covariates (STEC-serogroup, age, sex, region of residence, and season of infection), to classify them as hospital-acquired or not.

### Statistical analysis

Statistical analyses were conducted using Stata/IC, version 12.1 (StataCorp LP, College Station, TX, USA). Univariable analysis was conducted using Poisson regression with robust error variance to estimate risk ratios (RR) and 95% confidence intervals (CI) [36]. Logistic regression models were used to select covariates for adjusting the relationship of serogroups and hospitalization or death, respectively. Age was forced into the model because of its assumed independent effect on disease severity. A manual forward selection strategy was used for possible inclusion of further covariates. Selection was based on improvement of the logistic regression model assessed by the Bayesian information criterion (BIC) intending to achieve adequately fitting models while avoiding overfitting. The final main-effects logistic regression model was further investigated for statistical interactions between serogroups and adjustment variables using BIC. The variables selected for the final logistic regression models, were then used in a multivariable Poisson regression models with robust error variance to obtain risk ratios and associated CIs.

### Ethics statement

In Germany STEC and HUS notification data are collected within the legal framework of the Infection Protection Act [37]. The national notification database which is hosted by the Robert Koch Institute (RKI), Germany's federal level public health institute, is in essence a public use file (http://www3.rki.de/SurvStat/). Approval by an ethics committee and written consent, respectively, was not required because reporting data transmitted to the national notification database are anonymous.

## Results

### Study population

From 2004 through 2011, 12,587 STEC-illnesses were ascertained in the German reporting system. Except for 2011 where 4,909 STEC cases and 648 STEC-associated HUS were reported, annual reporting frequencies ranged from 834 to 1,180 STEC cases, corresponding to an incidence of 1 and 1.4 per 100,000, and from 32 to 60 cases of STEC-associated HUS (incidence <0.1 per 100,000).

Of the 12,587 reported STEC-illnesses, 579 cases were excluded from the analyses: 380 cases due to missing values in covariates or outcome variable, and 199 cases with potentially hospital-acquired infections (including 44 based on the imputation procedure). After preliminary analysis, we decided to exclude all cases with unknown O-antigen in 2011 (n = 3,608). The rational for this decision was that most of these illnesses likely belonged to a large outbreak of STEC O104:H4. Case-patients in 2011 with unknown STEC serogroup differed markedly from those in previous years. They were substantially older (median age 2011: 41 years vs. 2004–2010: 9 years), hospitalizations were twice as common (2011: 51% vs. 2004–2010: 24%) and more women were affected in 2011 (58%; 2004–2011: 51%). The majority of these cases (n = 2,392) met the case definition of the STEC O104:H4 outbreak [34]. Thus, including them would have largely determined the estimates for the group of case-patients for which the serogroup was not reported, thereby biasing the results.

Overall, 8,400 cases, incl. 627 (7%) HUS cases were analyzed. Median age of cases from 2004 through 2010 was 8 years (interquartile range [IQR], 2–43 years), and for 2011, 34 years (IQR, 12–56 years). Slightly more reported cases were female (53%), particularly in adults as of 20 years of age (62%).

For 4,325 cases (51%), 3,793 STEC and 532 HUS cases, serotyping results were reported. Serotyping information was significantly more frequently reported for HUS-cases (85%) than for STEC gastroenteritis cases (49%, p-value of chi-squared test <0.001). STEC belonged to 106 different serogroups (including O-non-typable and O-rough) with O104 as most frequently reported O-antigen (exclusively in 2011), followed by O157 (table 1). Except for 2011, serogroup distribution was fairly constant over the years with O157 being the most frequently reported serogroup, but accounting for less than 20% of the cases with available serogroup information. Case-patients' age varied markedly among serogroups. Median age of cases infected by STEC of serogroups O157, O26, O103, O111, and O145 was <5 years, whereas that of O128, O104 and O91 was ≥18 years.

**Table 1:** Characteristics of STEC-illnesses reported in Germany 2004-2001 –stratified by STEC serogroup.

		Male sex	Age*	Hospitalizations	Fatalities	HUS
Serogroup	No. of patients	No. of patients (%)	Median (IQR)	No. of patients (%)	No. of patients (%)	No. of patients (%)
O157	721	338 (46.9)	3 (1–10)	333 (46.2)	9 (1.2)	189 (26.2)
O104	917	336 (36.6)	44 (29–62)	623 (67.9)	11 (1.2)	264 (28.8)
O26	514	278 (54.1)	1.5 (1–4)	94 (18.3)	1 (0.2)	31 (6.0)
O103	426	215 (50.5)	2 (1–6)	48 (11.3)	0 (0.0)	3 (0.7)
O91	323	132 (40.9)	23 (6–48)	61 (18.9)	0 (0.0)	1 (0.3)
O145	190	84 (44.2)	2 (1–7)	45 (23.7)	1 (0.5)	15 (7.9)
O128	106	49 (46.2)	18 (2–40)	25 (23.6)	0 (0.0)	1 (0.9)
O111	100	51 (51.0)	2 (1–4)	21 (21.0)	0 (0.0)	10 (10.0)
Other	1,028	474 (46.1)	8 (1–39)	214 (20.8)	1 (0.1)	18 (1.8)
Unknown	4,075	1,994 (48.9)	9 (2–45)	990 (24.3)	7 (0.2)	95 (2.3)
Total	8,400	3,951 (47.0)	8 (2–43)	2,454 (29.2)	30 (0.4)	627 (7.5)

Percentages are calculated within serogroup categories;

* in years.

Abbrevations: IQR – interquartile range.

Among the 8,400 cases in this study, 2,454 (29%) hospitalizations were observed. The proportion of hospitalized cases varied across STEC serogroups and ranged from 11% for O103 to 68% for O104 (46% for O157) and across age-groups; it was lowest in children <3 years (21%) and highest in case-patients >65 years (53%, table 2).

**Table 2:** Number of STEC-illnesses reported in Germany 2004–2011 and percentage of hospitalized case-patients – by age-group and serogroup.

	Age group (years)											
	<3		3–9		10–18		19–40		41–65		>65	
Serogroup	No.	% hosp	No.	% hosp	No.	% hosp	No.	% hosp	No.	% hosp	No.	% hosp
O157	303	41.9	222	49.5	52	65.4	48	29.2	53	39.6	43	62.8
O104	8	50.0	23	78.3	68	70.6	300	66.0	327	63.9	191	76.4
O26	344	16.6	91	17.6	12	41.7	21	14.3	33	18.2	13	53.8
O103	243	8.6	89	9.0	18	11.1	29	13.8	28	28.6	19	26.3
O91	45	8.9	62	17.7	42	28.6	72	12.5	61	14.8	41	39.0
O145	108	21.3	43	16.3	12	58.3	10	20.0	10	20.0	7	57.1
O128	33	27.3	11	27.3	10	40.0	27	18.5	15	6.7	10	30.0
O111	68	17.6	16	31.3	5	20.0	3	33.3	5	20.0	3	33.3
Other	360	20.0	173	17.3	67	11.9	184	13.0	142	24.6	102	44.1
Unknown	1,314	19.9	771	19.6	269	29.7	579	17.1	634	24.1	508	48.2
Total	2,826	20.9	1,501	23.9	555	36.2	1,273	28.2	1,308	34.0	937	53.3

Percentages are calculated within serogroup categories.

30 deaths (0.4%) with a (co-)causal contribution of an STEC infection were ascertained in the surveillance system with a patient's median age of 60 years (IQR 2–82 years, table 3). The advanced median age was largely driven by STEC O104 cases, which accounted for the largest number of deaths (n = 11), followed by STEC O157 (n = 9). These two serogroups had the highest case-fatality ratio (1.2% each). Most cases for which death was recorded (n = 21) had HUS (case fatality: 3.4% compared to 0.1% for STEC-gastroenteritis), e.g., all fatal cases of STEC O157 had HUS (n = 9). Nearly 1/4 of fatalities (n = 8) was found in children <3 years (all had HUS), but almost half (n = 14) occurred in the highest age category (>65 years), of which most were infected by STEC O104.

**Table 3:** Deaths among reported STEC-illnesses in Germany 2004–2011 - by serogroup.

	Fatalities	Age*	Underlying HUS
Serogroup**	No. of patients	Median (IQR)	No. of patients (%)
O157	9	4 (1–6)	9 (100)
O104	11	80 (62–87)	7 (64)
O26	1	0	0 (0)
O145	1	73	0 (0)
Other***	1	79	0 (0)
Unknown	7	7 (0–82)	5 (71)
Total	30	60 (2–82)	21 (70)

Percentages are calculated within serogroup categories,

* in years.

** no fatalities were reported for infections with STEC O91, O103, O111, and O128.

*** serogroup of the fatal case in this category was reported as Ont (non-typable O antigen).

Abbrevations: HUS – hemolytic-uremic syndrome, IQR – interquartile range.

### Risk analysis

#### Hospitalization

In univariable analysis, only cases infected by O104 had a higher risk of hospitalization than those infected by O157 (table 4). Hospitalization risks of patients infected with other non-O157 serogroups were consistently and markedly lower. The average hospitalization risk for a patient infected by a non-O157 STEC (except O104) was less than half that of STEC O157. A multivariable model, adjusted for case-patients' age and residence, did not change the rank order among the serogroups and changed risk ratio-estimates only slightly (table 4). Statistical interaction terms of the different covariates did not improve the BIC of the model. The risk of hospitalization for case-patients infected by STEC O104 was 33% higher than that of STEC O157 (RR, 1.33; CI, 1.19–1.45). The RR of the other non-O157 STEC ranged from 0.54 for O145 to 0.27 for serogroup O103.

**Table 4:** Serogroup specific risk ratios for hospitalization among STEC-illnesses reported in Germany 2004–2011.

	Univariable model	Multivariable model*
Serogroup	RR (95% CI)	RR (95% CI)
O157	Reference	Reference
O104	1.47 (1.34–1.61)	1.33 (1.19–1.45)
O145	0.51 (0.39–0.67)	0.54 (0.41–0.70)
O128	0.51 (0.36–0.73)	0.49 (0.35–0.69)
Unknown	0.53 (0.48–0.58)	0.48 (0.44–0.53)
O111	0.45 (0.31–0.67)	0.48 (0.33–0.71)
Other	0.45 (0.39–0.52)	0.44 (0.38–0.51)
O26	0.40 (0.32–0.48)	0.43 (0.35–0.52)
O91	0.41 (0.32–0.52)	0.38 (0.30–0.48)
O103	0.24 (0.18–0.32)	0.27 (0.20–0.35)
Non-O157/O104**		0.41 (0.37–0.46)***

Risk ratios were calculated using Poisson regression with robust error estimate.

* adjusted for age and region of cases' residence.

** all except serogroups O157 and O104.

*** calculated in a separate model (serogroup categories: O157, non-O157/O104, O104, unknown).

Abbrevations: RR –risk ratio, CI – confidence interval.

#### Mortality

Age-adjusted mortality risk (table 5) was highest for patients infected by STEC O157 and O104 with no statistically significant difference between them (RR, 0.89; CI, 0.32–2.49). The risk for patients infected by any conventional non-O157 STEC was less than 10% compared with O157 (RR, 0.09; CI, 0.02–0.32). None of the other explanatory variables improved the BIC of the model.

**Table 5:** Serogroup specific risk ratios for mortality among STEC-illnesses reported in Germany 2004–2011.

	Univariable model	Multivariable model*
Serogroup	RR (95% CI)	RR (95% CI)
O157	Reference	Reference
O104	0.96 (0.40–2.31)	0.89 (0.32–2.49)
Unknown	0.14 (0.05–0.37)	0.13 (0.05–0.35)
Non-O157/O104**	0.09 (0.02–0.33)	0.09 (0.02–0.32)

Risk ratios were calculated using Poisson regression with robust error estimate.

* adjusted for age.

** all except serogroups O157 and O104.

Abbrevations: RR – risk ratio, CI – confidence interval.

## Discussion

We used nationwide reporting data from Germany to assess differences among STEC serogroups with regard to virulence - measured by hospitalization and death – irrespective of patients' age. This study yielded three main findings: First, cases infected by STEC O157, the most prominent STEC serogroup worldwide, were markedly more likely to be hospitalized or die compared to those infected by any of the non-O157 serogroups - except O104. Second, the investigated non-O157 STEC serogroups (excluding O104) varied in their virulence, although the difference across those non-O157 serogoups was less pronounced than the difference between single non-O157 serogroups and STEC O157. Third, case-patients infected by STEC O104, all part of one large outbreak in 2011, had a 1/3 higher risk of hospitalization and a comparable risk of dying than those infected by O157, according to German surveillance data.

These population-based data provide evidence for the exceptional virulence of O157 STEC, which accords with smaller studies that found a higher proportion of cases with bloody diarrhea or HUS in patients infected by STEC O157 than non-O157 STEC [13], [20], [21], [30], [38]. The presence of a rare sorbitol-fermenting clone of STEC O157:H- in Germany, which is believed to be of heightened virulence and seldom found in other countries yet, may have contributed to the observed difference in virulence between O157 and non-O157 STEC. However, sorbitol-fermenting STEC O157:H- is rarely diagnosed in patients without HUS [39] and most STEC O157 isolated in Germany from HUS patients in the study period belonged to sorbitol-nonfermenting strains. Therefore the impact of this clone to the overall virulence of serogroup O157 in this study likely is small. Patients infected by a non-O157 STEC (excluding O104) had, on average, less than half the risk to become hospitalized and 1/10 the risk of dying compared with O157-infected case-patients. The lowest hospitalization risk, only 1/4 that of O157, was found for patients infected by STEC O103, a serogroup frequently isolated from STEC gastroenteritis patients in Germany. Differences in hospitalization risks among the most frequently typed non-O157 serogroups were smaller than their risk compared to O157. Reported hospitalization is a fairly crude proxy for disease severity and other considerations, e.g. precautionary aspects, may contribute to the decision to hospitalize a patient. Therefore, collecting more detailed clinical markers of disease severity, e.g., duration of illness, severity (e.g., visible blood in stool) and frequency of loose stools may permit a more accurate characterization of disease severity, and thereby to better differentiate between the virulence of non-O157 serogroups.

Patients infected by STEC O104 belonged to the largest ever documented HUS outbreak that occurred in 2011. The causative agent, an *E. coli* O104:H4, combined virulence traits of STEC and of enteroaggregative *E. coli* [34], [40], [41]. A comparatively high virulence has been attributed to this strain based on the high proportion of HUS ascertained in this outbreak [34], [42]. This proportion could be corroborated in two additional studies that observed closed groups of people, i.e., in studies without the risk of disproportionately ascertaining severe cases. Yet, cases infected by STEC O104, unprecedentedly even HUS cases, were mostly adults, predominantly women. Furthermore, clinical courses of pediatric O104 HUS-patients did not differ from those infected by other STEC [43]. Host factors may have also contributed, at least partially, for the severity of this outbreak [44]. Of note, our comparison accounts for the most important (proxies for) host characteristics, age and sex, and revealed that the virulence of STEC O104(:H4) is comparable with that of STEC O157. We caution, however, for over-interpreting these data. Hospitalization risk was influenced by the heightened awareness and anxiety of gastroenteritis patients during the outbreak period, which likely led to a more liberal hospitalization policy of physicians in primary care and in hospitals.

Our study is subject to potential biases and limitations. We used national surveillance data, i.e., STEC-illnesses that were reported to local health departments (and transmitted to RKI via health authorities at federal state level). Ascertainment of patients' hospitalization status or death is likely incomplete, particularly if death occurred after local health departments finished their case investigation. Furthermore, reported illnesses may not be representative for all cases occurring in the German population. Nonetheless, our comparisons should still be valid under the proviso that serotyping was conducted independent from the decision to hospitalize the patient or from its death. Serotyping results were available for only 51.5% of all reported cases. This proportion was not substantially higher in hospitalized cases (and deceased cases), indicating that strain serotyping was not initiated more frequently in cases with severe outcomes – with one exception: Typing information for STEC isolated from gastroenteritis cases (without HUS) was less complete than for those from HUS patients. Consequently, investigated serogroups disproportionately contained more severe, i.e., HUS-associated, cases and STEC O157 was the serogroup most frequently found in STEC from HUS patients (except O104). As a result, the magnitude of the difference found between O157 and conventional non-157 serogroups with regard to hospitalization and death may have been overestimated by this study. However, as HUS cases represented only 7% of the study cases, the extent of this bias appears to be limited. Parenthetically, the disproportionate lack of typing information in STEC gastroenteritis cases should be considered when interpreting the ability of serogroups to cause HUS; likely the proportion of HUS cases among serogroup-specific illnesses are overestimated (except for O104).

The serogroup antigens, lipopolysaccharides on the bacterial surface, serve as a proxy for the genomic content of the strain. When coupled with information on virulence genes, particularly Shiga toxin genotype (*stx*), a more accurate assessment of the strains virulence can be achieved. Indeed, even among STEC O157 there are differences in the potential to cause HUS, depending on the strains' *stx* profile [21], [45]. Particularly *stx*_2_ is associated with severe disease [46], [47], but differences exist among subtypes of *stx*_2_ [45], [48]. Thus, a diagnostic procedure that detects in one diagnostic step the relevant *stx*-subtypes together with the *eae*-gene (and, ideally, the most important HUS serogroups) would allow a quick assessment of the strains' likely potential to cause HUS. Unfortunately, current diagnosis in primary care laboratories does not provide such information. Time is of the essence in the management of patients with acute bloody diarrhea (of all ages) [49], a symptom frequently caused by virulent STEC strains. Early intravenous volume expansion during diarrhea is associated with relative nephroprotection during subsequent pediatric HUS [50]. Furthermore, risk of secondary STEC O157 transmission is considerable, occurs early in the course of illness and can be markedly reduced by prompt spatial isolation of vulnerable contacts or of the primary patient [51].

Fortunately, presumptive identification of sorbitol-nonfermenting STEC O157 can be achieved within 24 hours after specimen receipt [50]. Thus, as long as primary care diagnostics do not provide the relevant information on the strains' virulence profile, we recommend using selective and differential media for STEC O157 diagnosis (coupled with nonculture STEC assays for detecting non-O157 STEC [19]) for stools submitted from persons with acute community-acquired diarrhea to enable timely identification of the most important subgroup of HUS-associated *E. coli* – sorbitol-nonfermenting STEC O157.

## References

[1] Donnenberg MS, Whittam TS (2001) Pathogenesis and evolution of virulence in enteropathogenic and enterohemorrhagic Escherichia coli. J Clin Invest 107: 539–548. 11238553 10.1172/JCI12404 PMC199431

[2] Ogura Y, Ooka T, Iguchi A, Toh H, Asadulghani M, et al (2009) Comparative genomics reveal the mechanism of the parallel evolution of O157 and non-O157 enterohemorrhagic Escherichia coli. Proc Natl Acad Sci U S A 106: 17939–17944. 19815525 10.1073/pnas.0903585106 PMC2764950

[3] Souza MR, Klassen G, Toni FD, Rigo LU, Henkes C, et al (2010) Biochemical properties, enterohaemolysin production and plasmid carriage of Shiga toxin-producing Escherichia coli strains. J Clin Microbiol 105: 318–321. 10.1590/s0074-02762010000300013 20512247

[4] Mead PS, Griffin PM (1998) Escherichia coli O157:H7. Lancet 352: 1207–1212. 9777854 10.1016/S0140-6736(98)01267-7

[5] Werber D, Behnke SC, Fruth A, Merle R, Menzler S, et al (2007) Shiga toxin-producing Escherichia coli infection in Germany: different risk factors for different age groups. Am J Epidemiol 165: 425–434. 17158472 10.1093/aje/kwk023

[6] Banatvala N, Griffin PM, Greene KD, Barrett TJ, Bibb WF, et al (2001) The United States National Prospective Hemolytic Uremic Syndrome Study: microbiologic, serologic, clinical, and epidemiologic findings. J Infect Dis 183: 1063–1070. 11237831 10.1086/319269

[7] Verweyen HM, Karch H, Allerberger F, Zimmerhackl LB (1999) Enterohemorrhagic Escherichia coli (EHEC) in pediatric hemolytic-uremic syndrome: a prospective study in Germany and Austria. Infection 27: 341–347. 10624594 10.1007/s150100050040

[8] March SB, Ratnam S (1986) Sorbitol-MacConkey medium for detection of Escherichia coli O157:H7 associated with hemorrhagic colitis. J Clin Microbiol 23: 869–872. 3519658 10.1128/jcm.23.5.869-872.1986 PMC268739

[9] Karmali MA (1989) Infection by verocytotoxin-producing Escherichia coli. Clin Microbiol Rev 2: 15–38. 2644022 10.1128/cmr.2.1.15 PMC358098

[10] Bettelheim KA (2007) The non-O157 shiga-toxigenic (verocytotoxigenic) Escherichia coli; under-rated pathogens. Crit Rev Microbiol 33: 67–87. 17453930 10.1080/10408410601172172

[11] Brooks JT, Sowers EG, Wells JG, Greene KD, Griffin PM, et al (2005) Non-O157 Shiga toxin-producing Escherichia coli infections in the United States, 1983–2002. J Infect Dis 192: 1422–1429. 16170761 10.1086/466536

[12] Werber D, Beutin L, Pichner R, Stark K, Fruth A (2008) Shiga toxin-producing Escherichia coli serogroups in food and patients, Germany. Emerg Infect Dis 14: 1803–1806. 18976578 10.3201/eid1411.080361 PMC2630736

[13] Gould LH, Mody RK, Ong KL, Clogher P, Cronquist AB, et al (2013) Increased recognition of non-O157 Shiga toxin-producing Escherichia coli infections in the United States during 2000–2010: epidemiologic features and comparison with E. coli O157 infections. Foodborne Pathog Dis 10: 453–460. 23560425 10.1089/fpd.2012.1401

[14] Fey PD, Wickert RS, Rupp ME, Safranek TJ, Hinrichs SH (2000) Prevalence of non-O157:H7 shiga toxin-producing Escherichia coli in diarrheal stool samples from Nebraska. Emerg Infect Dis 6: 530–533. 10998385 10.3201/eid0605.000513 PMC2627960

[15] Lockary VM, Hudson RF, Ball CL (2007) Shiga toxin-producing Escherichia coli, Idaho. Emerg Infect Dis 13: 1262–1264. 17953111 10.3201/eid1308.070189 PMC2828089

[16] Jelacic JK, Damrow T, Chen GS, Jelacic S, Bielaszewska M, et al (2003) Shiga toxin-producing Escherichia coli in Montana: bacterial genotypes and clinical profiles. J Infect Dis 188: 719–729. 12934188 10.1086/376999

[17] Buvens G, De Gheldre Y, Dediste A, de Moreau AI, Mascart G, et al (2012) Incidence and virulence determinants of verocytotoxin-producing Escherichia coli infections in the Brussels-Capital Region, Belgium, in 2008–2010. J Clin Microbiol 50: 1336–1345. 22238434 10.1128/JCM.05317-11 PMC3318570

[18] Wylie JL, Van Caeseele P, Gilmour MW, Sitter D, Guttek C, et al (2013) Evaluation of a new chromogenic agar medium for detection of Shiga toxin-producing Escherichia coli (STEC) and relative prevalences of O157 and non-O157 STEC in Manitoba, Canada. J Clin Microbiol 51: 466–471. 23175263 10.1128/JCM.02329-12 PMC3553920

[19] Gould LH, Bopp C, Strockbine N, Atkinson R, Baselski V, et al (2009) Recommendations for diagnosis of shiga toxin–producing Escherichia coli infections by clinical laboratories. MMWR Recomm Rep 58: 1–14. 19834454

[20] Hadler JL, Clogher P, Hurd S, Phan Q, Mandour M, et al (2011) Ten-year trends and risk factors for non-O157 Shiga toxin-producing Escherichia coli found through Shiga toxin testing, Connecticut, 2000–2009. Clin Infect Dis 53: 269–276. 21765075 10.1093/cid/cir377

[21] Hedican EB, Medus C, Besser JM, Juni BA, Koziol B, et al (2009) Characteristics of O157 versus non-O157 Shiga toxin-producing Escherichia coli infections in Minnesota, 2000–2006. Clin Infect Dis 49: 358–364. 19548834 10.1086/600302

[22] Rangel JM, Sparling PH, Crowe C, Griffin PM, Swerdlow DL (2005) Epidemiology of Escherichia coli O157:H7 outbreaks, United States, 1982–2002. Emerg Infect Dis 11: 603–609. 15829201 10.3201/eid1104.040739 PMC3320345

[23] Hermos CR, Janineh M, Han LL, McAdam AJ (2011) Shiga toxin-producing Escherichia coli in children: diagnosis and clinical manifestations of O157:H7 and non-O157:H7 infection. J Clin Microbiol 49: 955–959. 21177902 10.1128/JCM.02119-10 PMC3067718

[24] Johnson KE, Thorpe CM, Sears CL (2006) The emerging clinical importance of non-O157 Shiga toxin-producing Escherichia coli. Clin Infect Dis 43: 1587–1595. 17109294 10.1086/509573

[25] McPherson M, Lalor K, Combs B, Raupach J, Stafford R, et al (2009) Serogroup-specific risk factors for Shiga toxin-producing Escherichia coli infection in Australia. Clin Infect Dis 49: 249–256. 19522658 10.1086/599370

[26] Clogher P, Hurd S, Hoefer D, Hadler JL, Pasutti L, et al (2012) Assessment of physician knowledge and practices concerning Shiga toxin-producing Escherichia coli infection and enteric illness, 2009, Foodborne Diseases Active Surveillance Network (FoodNet). Clin Infect Dis 54 Suppl 5: S446–452. 22572668 10.1093/cid/cis246

[27] Stigi KA, Macdonald JK, Tellez-Marfin AA, Lofy KH (2012) Laboratory practices and incidence of non-O157 shiga toxin-producing Escherichia coli infections. Emerg Infect Dis 18: 477–479. 22377034 10.3201/eid1803.111358 PMC3309587

[28] Buvens G, Pierard D (2012) Virulence profiling and disease association of verocytotoxin-producing Escherichia coli O157 and non-O157 isolates in Belgium. Foodborne Pathog Dis 9: 530–535. 22545959 10.1089/fpd.2011.1073

[29] Blanco JE, Blanco M, Alonso MP, Mora A, Dahbi G, et al (2004) Serotypes, virulence genes, and intimin types of Shiga toxin (verotoxin)-producing Escherichia coli isolates from human patients: prevalence in Lugo, Spain, from 1992 through 1999. J Clin Microbiol 42: 311–319. 14715771 10.1128/JCM.42.1.311-319.2004 PMC321739

[30] Klein EJ, Stapp JR, Clausen CR, Boster DR, Wells JG, et al (2002) Shiga toxin-producing Escherichia coli in children with diarrhea: a prospective point-of-care study. J Pediatr 141: 172–177. 12183710 10.1067/mpd.2002.125908

[31] Lathrop S, Edge K, Bareta J (2009) Shiga toxin-producing Escherichia coli, New Mexico, USA, 2004–2007. Emerg Infect Dis 15: 1289–1291. 19751594 10.3201/eid1508.08151515 PMC2815966

[32] Manning SD, Madera RT, Schneider W, Dietrich SE, Khalife W, et al (2007) Surveillance for Shiga toxin-producing Escherichia coli, Michigan, 2001–2005. Emerg Infect Dis 13: 318–321. 17479902 10.3201/eid1302.060813 PMC2725841

[33] Gould LH, Demma L, Jones TF, Hurd S, Vugia DJ, et al (2009) Hemolytic uremic syndrome and death in persons with Escherichia coli O157:H7 infection, foodborne diseases active surveillance network sites, 2000–2006. Clin Infect Dis 49: 1480–1485. 19827953 10.1086/644621

[34] Frank C, Werber D, Cramer JP, Askar M, Faber M, et al (2011) Epidemic profile of Shiga-toxin-producing Escherichia coli O104:H4 outbreak in Germany. N Engl J Med 365: 1771–1780. 21696328 10.1056/NEJMoa1106483

[35] Cleveland WS (1979) Robust locally weighted regression and smoothing scatterplots. Journal of the American Statistical Association 74: 829–836.

[36] Zou G (2004) A Modified Poisson Regression Approach to Prospective Studies with Binary Data. Am J Epidemiol 159: 702–706. 15033648 10.1093/aje/kwh090

[37] Bundesrepublik_Deutschland (2000) Gesetz zur Verhütung und Bekämpfung von Infektionskrankheiten beim Menschen (Infektionsschutzgesetz - IfSG). Bundesgesetzblatt, Teil I, Nr. 33. pp. 1045–1077.

[38] Werber D, Fruth A, Heissenhuber A, Wildner M, Prager R, et al (2004) Shiga toxin-producing Escherichia coli O157 more frequently cause bloody diarrhea than do non-O157 strains. J Infect Dis 189: 1335–1336; author reply 1336–1337. 15031806 10.1086/382282

[39] Nielsen S, Frank C, Fruth A, Spode A, Prager R, et al (2011) Desperately seeking diarrhoea: outbreak of haemolytic uraemic syndrome caused by emerging sorbitol-fermenting shiga toxin-producing Escherichia coli O157:H-, Germany, 2009. Zoonoses Public Health 58: 567–572. 21824358 10.1111/j.1863-2378.2011.01405.x

[40] Brzuszkiewicz E, Thurmer A, Schuldes J, Leimbach A, Liesegang H, et al (2011) Genome sequence analyses of two isolates from the recent Escherichia coli outbreak in Germany reveal the emergence of a new pathotype: Entero-Aggregative-Haemorrhagic Escherichia coli (EAHEC). Arch Microbiol 193: 883–891. 21713444 10.1007/s00203-011-0725-6 PMC3219860

[41] Bielaszewska M, Mellmann A, Zhang W, Kock R, Fruth A, et al (2011) Characterisation of the Escherichia coli strain associated with an outbreak of haemolytic uraemic syndrome in Germany, 2011: a microbiological study. Lancet Infect Dis 11: 671–676. 21703928 10.1016/S1473-3099(11)70165-7

[42] King LA, Nogareda F, Weill FX, Mariani-Kurkdjian P, Loukiadis E, et al (2012) Outbreak of Shiga toxin-producing Escherichia coli O104:H4 associated with organic fenugreek sprouts, France, June 2011. Clin Infect Dis 54: 1588–1594. 22460976 10.1093/cid/cis255

[43] Loos S, Ahlenstiel T, Kranz B, Staude H, Pape L, et al (2012) An outbreak of Shiga toxin-producing Escherichia coli O104:H4 hemolytic uremic syndrome in Germany: presentation and short-term outcome in children. Clin Infect Dis 55: 753–759. 22670043 10.1093/cid/cis531

[44] Werber D, King LA, Muller L, Follin P, Buchholz U, et al (2013) Associations of Age and Sex With the Clinical Outcome and Incubation Period of Shiga toxin-producing Escherichia coli O104:H4 Infections, 2011. Am J Epidemiol 10.1093/aje/kwt069 23935124

[45] Soborg B, Lassen S, Muller L, Jensen T, Ethelberg S, et al (2013) A verocytotoxin-producing E. coli outbreak with a surprisingly high risk of haemolytic uraemic syndrome, Denmark, September–October 2012. Euro Surveill 18. 23324425

[46] Boerlin P, McEwen SA, Boerlin-Petzold F, Wilson JB, Johnson RP, et al (1999) Associations between virulence factors of Shiga toxin-producing Escherichia coli and disease in humans. J Clin Microbiol 37: 497–503. 9986802 10.1128/jcm.37.3.497-503.1999 PMC84443

[47] Ostroff SM, Tarr PI, Neill MA, Lewis JH, Hargrett-Bean N, et al (1989) Toxin genotypes and plasmid profiles as determinants of systemic sequelae in Escherichia coli O157:H7 infections. J Infect Dis 160: 994–998. 2685131 10.1093/infdis/160.6.994

[48] Friedrich AW, Bielaszewska M, Zhang WL, Pulz M, Kuczius T, et al (2002) Escherichia coli harboring Shiga toxin 2 gene variants: frequency and association with clinical symptoms. J Infect Dis 185: 74–84. 11756984 10.1086/338115

[49] Holtz LR, Neill MA, Tarr PI (2009) Acute bloody diarrhea: a medical emergency for patients of all ages. Gastroenterology 136: 1887–1898. 19457417 10.1053/j.gastro.2009.02.059

[50] Hickey CA, Beattie TJ, Cowieson J, Miyashita Y, Strife CF, et al (2011) Early volume expansion during diarrhea and relative nephroprotection during subsequent hemolytic uremic syndrome. Arch Pediatr Adolesc Med 165: 884–889. 21784993 10.1001/archpediatrics.2011.152 PMC4064458

[51] Werber D, Mason BW, Evans MR, Salmon RL (2008) Preventing household transmission of Shiga toxin-producing Escherichia coli O157 infection: promptly separating siblings might be the key. Clin Infect Dis 46: 1189–1196. 18444854 10.1086/587670

